# Reviews

**Published:** 1922-02

**Authors:** 


### REVIEWS OF BOOKS.

**THE D'HERELLE PHENOMENON AND THE TRANSMISSION OF ACQUIRED PROPERTIES,** from the Bulletin of the Johns Hopkins Hospital, No. 367.

Interest in the transmission of acquired properties has been again aroused by what is known as the d'Herelle phenomenon. A good account of this is given in the report of a lecture by Professor Jules Bordet at the Johns Hopkins Hospital, and published in the Johns Hopkins Hospital Bulletin, No. 367. Dysentery bacilli, submitted to the action of leucocytes derived from an animal immunised against dysentery bacilli, or from the fæces of a patient recovering from an attack of dysentery, acquire the power of dissolving bacteria not so treated, and can transmit this property to their descendants.

In considering this subject it is important to distinguish between conditions which act on bacteria as members of the general body of living organisms (such conditions being temperature, oxygen supply, food supply, reaction as regards acidity of the medium in which they live, &c.), and specific conditions or stimuli, which select certain bacteria, and also certain properties of these bacteria, on which to act. The former may be compared, in the case of animals, to the action of food and climate, and the latter to the action of diphtheria toxin on certain animals, and on certain organs of these animals.

In the case of bacteria the specific stimulus can be furnished by an antiserum, the action of which is shown by agglutination, &c., of the corresponding bacterium. Speaking generally, the protoplasm of bacteria possesses certain properties such as reproduction, the power to digest particular classes of matter, and the power to excite the production of an anti-body, specific for each variety, when injected into a living animal.

Since bacteria are unicellular organisms, and during at least part of their life there is little or no differentiation of their protoplasm, it is reasonable to assume that all these properties are possessed in common by the protoplasm.

If we can apply and confine a stimulus to one of these qualities we may expect that the protoplasm which grows from, or is reproduced from, that which is thus stimulated, will show this quality, accentuated or diminished according as the stimulus was of a moderate strength, sufficient to act as a tonic; or of excessive strength, capable of producing paralysis, or destruction, of the special quality. We can, in fact, by means of an antiserum, apply a stimulus to a particular quality, or group of qualities, of a bacterium, such as its virulence.

What has been said offers an explanation of the d'Herelle phenomenon and of the results of experiments on the transmission of acquired properties in bacteria described previously by other workers.

In the higher animals the various properties, or qualities, are differentiated into certain specialised cells, grouped into agglomerations called organs. Consequently, it is unlikely if we produce a serum anti to muscle or pancreas cells, for example, that we shall, if we inject the serum into an animal, affect the reproductive cell in such a manner that the descendants of the animal will show qualities connected with muscle or pancreas, increased or diminished. The process of separation of the qualities, that is differentiation, has gone so far that the antiserum and the reproductive cells have no points of correspondence with each other. To produce a change in the descendants we allow the reproductive cells from nearly allied animals to fuse. In fact, we cross-breed. We make use also of chance variations in an animal of the same species to select a partner to breed with; or, we select animals with which to breed, possessing certain qualities, in order to have these preserved and intensified in their descendants. But this is very different from what can be done in the case of bacteria, where we can readily impress a quality on a bacterium by using a stimulus specific for this quality, and insure its transmission to its descendants.

The problem in the case of the higher animals is extraordinarily difficult, but, we may hope, not insoluble. Chance variations do occur. This, however, means that variations can be produced, but we do not know how to produce them at will. It is to be hoped and expected that the factor or factors which produce variations will be discovered and that control of these will be obtained.—A. R. F.

**THE PHYSIOLOGY OF GOUT, RHEUMATISM, AND ARTHRITIS.** By Percy Wilde. Pp. 220. (John Wright & Sons, Ltd., Bristol, and Simpkin, Marshall & Co., Ltd.; 12s. 6d. net.)

St. Paul advised his young disciple to "prove all things." Dr. Wilde has taken his advice and has proved all things wrong. The reader is left with no doubt in his mind of the absolute sincerity with which the author's views are put forward. He has studied his problem for many years, has thought, has experimented, and has convinced himself that the whole of the accepted views as to the nature of Gout, Rheumatism and Chronic Arthritis are false and founded on error. His own views are either "very wrong" or "very right"; in either case they stimulate thought, and the clinical results that he describes compel admiration. This is no book for the undergraduate (it is far too rebellious against authority), but it will repay careful reading by the man who, having passed his examination-days, is allowed to think for himself. To such an indication of the thesis may be given in the form of a few statements based upon the author's observations: Uric acid is never present in body-tissues or body-fluids; "urate of soda" is not a urate at all, but a combination of urea and sodium chloride; tophi are not composed of urates; the familiar diagrams of uric acid crystals do not represent uric acid, but lactophosphate of lime; chronic rheumatism is caused by prolonged and repeated exposure to cold; it is not due to "infection," and vaccines are of value only in so far as they cause a rise of temperature. On his view that chemical changes can be brought about by purely

**Reviews of Books**—(*cont.*).

physical changes the author bases his line of "pyretic" treatment, which he describes in detail. The profession cannot accept the views here set forth without careful study of the problems involved; but the book leaves an impression of sincerity, of conviction on the part of its writer, which should encourage the critical study of his observations and suggestions. To italicise the author's own words—"neither recognised routine treatment *nor the results of the clinical experience of others can be accepted as a safe guide*."—C. E. S.

**CATARACT AND ITS TREATMENT.** By Henry Kirkpatrick, M.B., Lieut.-Colonel, I.M.S. Pp. xiii., 201, with 70 illustrations. (Henry Frowde and Hodder & Stoughton. 12s. 6d. net.)

Cataract extraction is one of the major operations in ophthalmic surgery, and is perhaps the disease of the eye which possesses the greatest interest to the ophthalmic surgeon. The removal of cataract is a highly satisfactory operation, both to the surgeon and to the patient. The Civil Surgeon in India gets a unique experience in the extraction of cataract and possesses exceptional opportunities for the investigation into the causation and best means of extraction of cataract. There are many variations in the ways of extracting cataracts; those practised in the East differ from those practised in the West, and both methods are themselves subject of wide variations. Much work has been done recently in regard to cataract, although the treatment of cataract is at the moment purely surgical. Doubtless in time when we have a true insight into the ætiological factors at work we shall be in a position to prevent the formation and keep stationary those which have developed.

This work, coming from the pen of a surgeon in the I.M.S. who has spent most of his service in India on ophthalmic appointments, ought to be of very great value. The book is divided into sixteen chapters, beginning with the anatomy of the lens and ending up with statistics and after-results. The descriptions of the operative proceedings are given in considerable detail, all the instruments being depicted. Equally fully given are the complications and their treatment. At the end of many of the chapters references are given for those who desire to pursue the subjects more fully.

This book is essentially practical, and ought to be of great value to all those who practice ophthalmic surgery, but more specially should it appeal to the surgeon in India who has frequent changes of station, as it contains in small volume the essential facts in sufficient detail.—T. P. N.

**THOUGHTS IN HOSPITAL.** By Lawrence Pilkington. (Longmans, Green; 2s. 6d. net.)

It is a pleasure to meet a volume, especially one in verse, by a hospital patient upon his experience in the ward which is neither silly, nor sentimental, nor betraying a lack of any sense of style. Oddly enough, as we have often lamented, the hospital, unlike such sister institutions as the school, has provided little genuine literature. Whitman's "Specimen Days," Henley's verses, an allusion or two by the present Poet Laureate, almost exhaust the list; the remainder is typified, generously typified, by Tennyson's sentimental lines which seem to have set their stamp upon a vulgar literary fashion. Even Mr. Compton Leith, from whom better things were expected by his admirers, produced in "Domus Doloris" only a regrettable exercise in preciosity and mannerism. We therefore turned to Mr. Lawrence Pilkington's modest, and charmingly printed, little volume with a faint, but inextinguishable, hope of something better. His aim is to give in a few hundred lines of blank verse, relieved by a few unequal lyrics, the sensations of a hospital patient during, apparently, convalescence after a surgical operation. Though he was, perhaps, extremely lucky in his sisters and nurses, Mr. Pilkington, in agreeable verse, has an eye, and observation. He notes (what patient in a London hospital can fail to note? but how few record!) the noise of the trams, of bells, of whistles, of the passing traffic, and the hum, almost the refrain, which these make through the slow night hours of broken sleep and troubled dreams. Indeed, his verses are by way of a comment to an imaginary "musical setting"; the "harps" and an "orchestra" are invoked to fill the gaps. Technically this is an error, because the poet must make his own music, if there is to be music at all. Some of the verse, however, is musical, and there is a strain of genuine insight and poetical expression in such lines as:—

> As in a dream it comes again to me,
> How first I saw the glories of the Alps
> When passing Basle I entered Switzerland
> And saw, in the grey light of early morn,
> Low, verdant hills with little firwoods crowned,
> Threaded with mist, the valleys full of kine
> Chewing the scented meadow-grass, their bells
> Sweet sounding. . . .
> The pearly, snow-clad mountains of the Alps.

That is a true picture; and "pearly" well expresses the soft, round curves of the Swiss snow, which curves do have the curious effect of changing the tone of the snow from dead white to pearl colour.

An admirable line is:—

> My pillow is an ass, and on its back
> I'm riding. . . .

also this:—

> I like to hear the deep, vibrating note
> Come booming from the distant town-hall clock;
> I like still more the harsh, insistent stroke
> With which the neighbouring church bell marks each hour.

Only people who have never been ill imagine that a harsh-throated clock annoys a sick patient. To mark the passage of time, however hoarsely, is a friendly act to aching nerves. Mr. Pilkington incidentally pays a tribute of gratitude to the voluntary system. If he ends too many lines with adjectives, he has a radicle of genuine poetry in him. He touches, only half successfully, on the subject of pain. Does he know Coventry Patmore's very remarkable ode upon the subject? But we are grateful for "Thoughts in Hospital"; and every doctor, secretary or nurse who cares for letters should insist on the addition of this volume to that uncultivated corner, unworthily boasting itself the hospital library!—O. B.

